# Dental Implants Acting as External Fixation for the Fracture of Severe Atrophic Mandible: A Case Report

**DOI:** 10.1007/s12663-023-02064-6

**Published:** 2023-11-30

**Authors:** H. Kinoshita, T. Ogasawara, T. Nishibata, M. Yoshioka, R. Makihara, Y. Hashimoto

**Affiliations:** Division of Dentistry and Oral Surgery, Fukui General Hospital, Egami, Fukui 910-8561 Japan

**Keywords:** Transmucosal fixation, Dental implant superstructure, Atrophic mandibular fracture, Edentulous mandibular fracture, Bony union

## Abstract

Treatment of edentulous and atrophic mandibular fractures is extremely difficult. Generally, mandibular fractures are repaired and fixed as internal fixation using a reconstruction plate or miniplates with intra- or extraoral approach. Few cases in which external fixation including a transmucosal fixation was performed have also been reported. We report a case of atrophic and edentulous mandibular fracture which was healed by the fixation using dental implants and implant-supported bridge.

Mandible atrophy results from tooth loss, its vertical height gradually decreases [1]. Severe edentulous atrophic mandible is prone to fractures, and the treatment is difficult　[2]. In these cases, the miniplates and reconstructive plates are used to fix the bone fragment [3]. However, the failure of fixation may cause the malunion, nonunion or false joint [4]. We report a case of a severely atrophic edentulous mandible fracture in which the dental implant and implant-supported bridge acted as external fixation and bone union was obtained.

A 58-year-old male patient with severe swelling of the right mandible presented to our clinic. The patient had paralysis of the entire oral cavity and bilateral lower lips and surrounding skin, so he did not complain of pain despite the severely swelling with dysphagia due to the odontogenic infection. The examination of paralysis in oral region by neurologist was unable to determine the cause. Two times of such severe odontogenic infections occurred, and the mandible became edentulous due to the removal of unsaveable infected teeth. A full denture was made but it is difficult to eat satisfactory because of the severe atrophic mandible. Therefore, since the patient did not use a full denture, maxillary teeth injured entire gingiva of mandibular alveolar ridge. The patient did not complain of gingival pain during chewing because of the numbness of the oral cavity. We planned and suggested the dental implant treatment to the patient in view of avoiding the mandibular infection by maxillary teeth. The mandibular bone condition was inspected by CT scan, which revealed a fracture with bony displacement in the midline of the mandible. It was inferred that the fracture had occurred after the last visiting to our outpatient clinic. The open reduction and internal fixation using a miniplate was performed under general anesthesia. Two weeks after this surgery, CT scan was taken again for planning of dental implants placement. The vertical height of mandible was insufficient, and the apex of 7-, 8.5- and 10-mm long dental implant bodies in each appropriate implant sites of both side molar regions reached the mandibular canal on simulation. Considering the infection of the miniplate by the edge of mandibular full denture or maxillary teeth, dental implant treatment was the only way to obtain the proper chewing and to avoid similar odontogenic infection which occurred last time. Although the patient had paralysis in the oral cavity including the area innervated by the alveolar nerve, the informed consent about the possibility of further hypoesthesia due to the insertion of dental implants was given carefully. The patient wanted dental implant treatment after appropriate and accurate description concerning the risks of infection or mandibular fracture due to dental implant placement. We placed eight dental implant bodies without the contact miniplate and/or screws in the mandible under the general anesthesia (Fig. 1a and b). After this first-stage surgery, the soft prosthesis was made to protect the wound from the maxillary teeth. The post-implantation course was good, and a second-stage surgery, impression of the superstructure and occlusal sampling were performed 3 months after the first-stage surgery. All eight dental implant bodies were found osseointegration. The superstructure was made of a fixed screw type. However, the patient came to the outpatient clinic with a swollen left side mandible before medical appointment of superstructure mounting. According to the patient, he had fallen 10 days before which timing is between after taking an impression of superstructure and before setting the superstructure. Two dental implant bodies in the left side premolar region were dislodged, and CT image showed mandibular fracture in the same site (Fig. 2a–c). The bone cross-section of the fracture site was the smallest in the mandible (Fig. 2b and c). In addition, an external dental fistula was observed on the skin of the fracture site. It was suggested that a hard blow to the left side of the mandible resulted in the dental implant bodies dislocation, fracture and infection. The mandible was complete freely mobile at the fracture site. Therefore, the superstructure of the six remaining dental implant bodies could be screw fixed appropriately (Fig. 2a and d). The mandibular fragment was displaced, and the mobile mandible was fixed at the position with matching each abutment and implant surfaces. This superstructure served as an external fixation. Opening and closing mouths and chewing were performed without problems. One year after superstructure placement, CT image showed ossification of the fracture site (Fig. 3a–c). The external dental fistula also disappeared. Four years have passed since the superstructure was mounted, and the clinical progress is good. The patient is able to chew well without peri-implantitis.

**Fig. 1:**
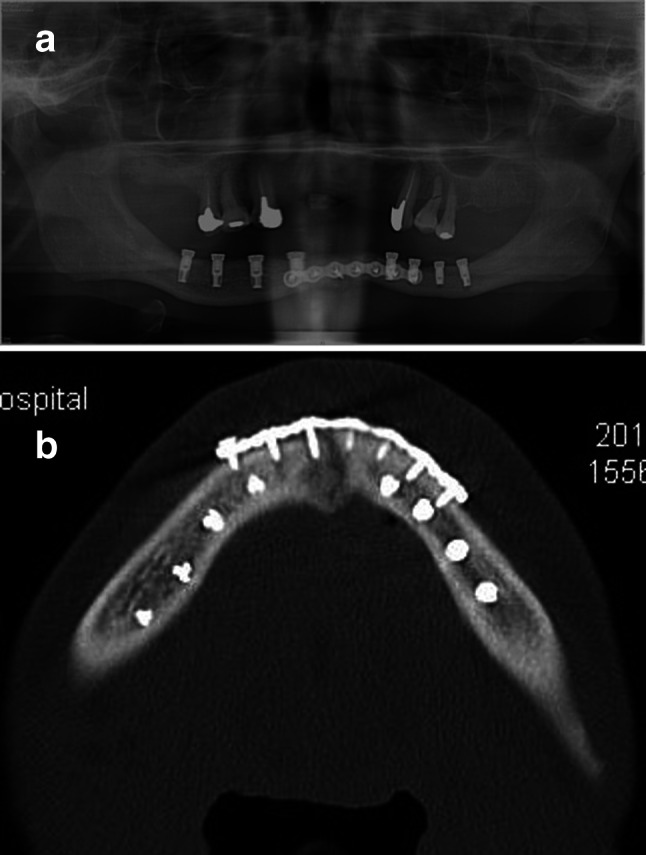
**a** Panoramic radiograph after implant bodies placement and **b** a fracture line is seen in the midline of the mandible on horizontal CT image. No contact between the implant bodies and the screws is allowed

**Fig. 2:**
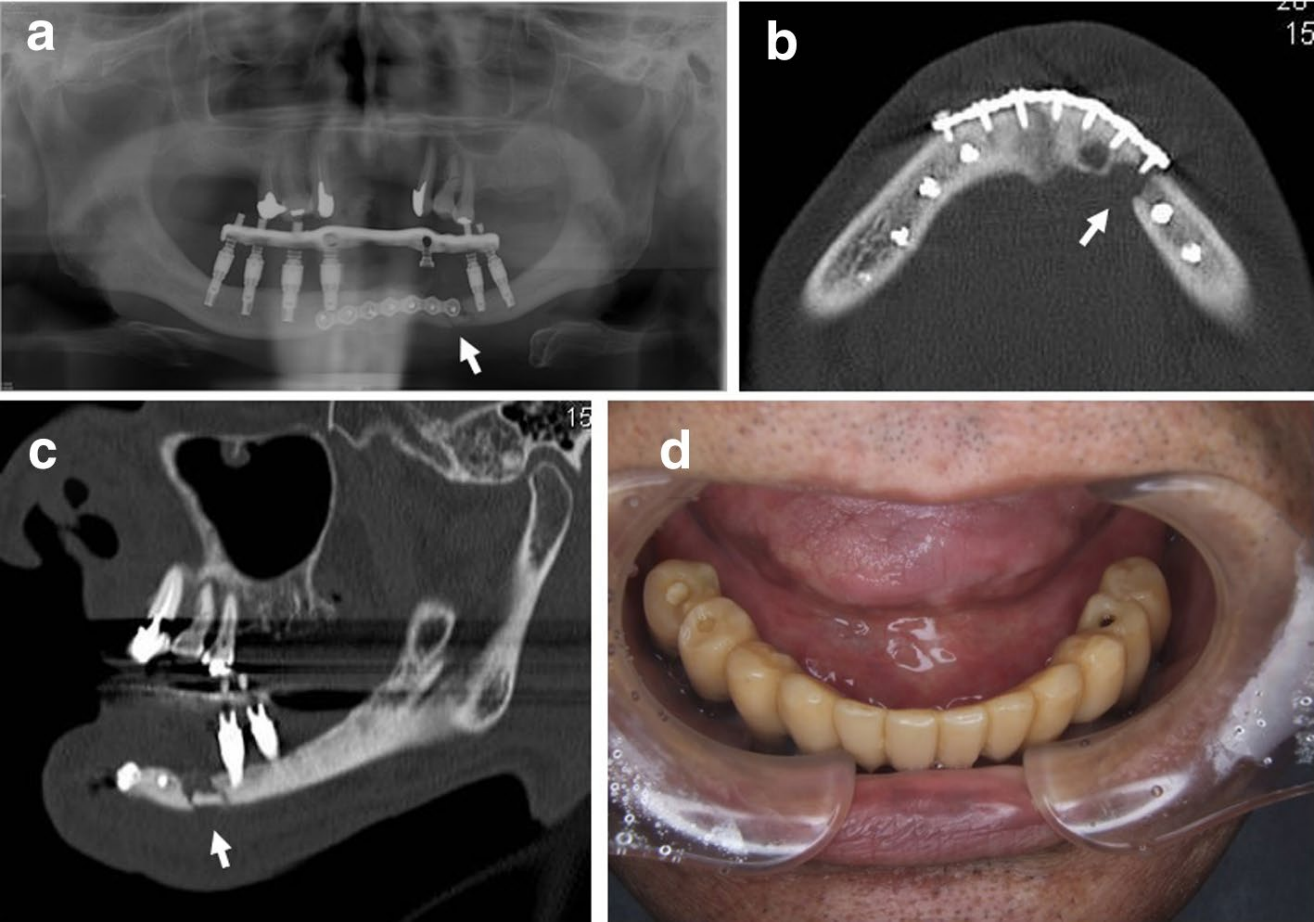
**a** Fracture ( →) in the left mandibular premolar on panoramic radiograph, **b** fracture ( →) on horizontal CT image, **c** the fracture site ( →) had the smallest bone height diameter on sagittal CT image and **d** intraoral photograph after the seating of superstructure

**Fig. 3:**
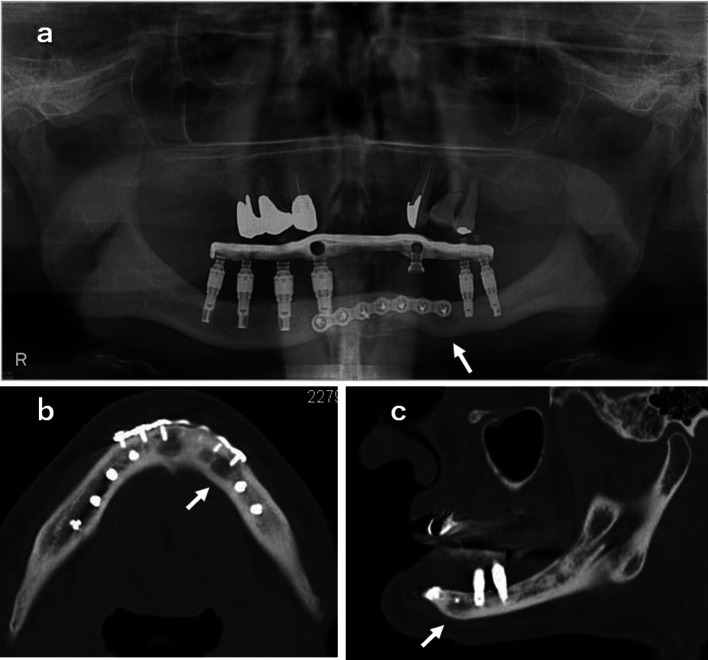
Bone fusion was observed 1 year after superstructure attachment at fracture site ( →) **a** panoramic radiograph, **b** horizontal CT image and **c** sagittal CT image

## Discussion

The treatment for repair of fractures of the severely atrophic and edentulous mandible is difficult. In many cases, the miniplates or reconstructive plates are used to fix bone fragment [3]. There is no consensus on the best treatment, especially in cases with a vertical height of less than 10 mm [5]. When the fixation was not done appropriately, it causes malunion and nonunion [4]. Also, considering denture-bearing area and the position of the plate is very important to avoid the infection and/or the plate exposure [6]. In our case, miniplate was fixed at the inferior margin side in the mandible to avoid infection and exposure of the miniplate due to the maxillary teeth against the mandible residual ridge. Therefore, the first fracture surgical procedure was selected extraoral approach without the intraoral incision lines.

A miniplate was selected as fixing device because of the large bone contact surface in the fracture site of mandibular median and the need for dental implant placement after this fracture surgery. The use of a reconstructive plates was thought to result in further fracture induced by the large screws and the contacts of dental implant bodies to be placed and screws of reconstructive plates. In this case, eight dental implant bodies could be placed without contact between dental implants bodies and screws of miniplate after restoration of the first mandibular median fracture.

The second mandibular fracture occurred after taking an impression of a dental implant superstructure. The fracture site had the smallest bone height diameter in the left side mandible. It was difficult to fixate the new fracture site with a miniplate or a reconstructive plate after removal of the existing miniplate in the midline of the mandible. External fixation of mandible fractures is a useful when an open surgery is difficult because of extensive comminution, bone or soft tissue loss and infection [7]. In particular, external fixation adopted to the mandible fracture by gunshot provides many advantages owing to its versatility and simplicity of use [8]. Transmucosal fixation is one strategy for the treatment of edentulous mandibular fracture using external fixation principles within the oral cavity [9]. There is a report of good healing results using this intraoral locking plate fixation technique for fractures of the edentulous mandible. Similar to these concepts, the mandibular fragment was fixed by attaching the superstructure to the residual dental implants in our case. Since the superstructure had already been created before the second fracture, it was possible to restore the mandible to its original position by attaching the superstructure. In addition, patients were able to chew from soft foods. It was reported that the duration of the external fixator usually varies from 8 to 12 weeks, sufficient time for bone repair and remodeling [10]. In our case, the complete bone union of fracture site was confirmed by CT about 1 year after seating of the superstructure. The patient was managed regularly with attention to peri-implantitis. Four years have passed since the superstructure was mounted, but the clinical progress is good. Further follow-up is required.
